# Regionalization of Health Care in Head and Neck Cancer: Concept and Considerations

**DOI:** 10.1055/s-0045-1802574

**Published:** 2025-08-20

**Authors:** Sebastián Castro, Mario Tapia, Felipe Cardemil

**Affiliations:** 1 Department of Otorhinolaryngology, Hospital San José, Santiago, Chile; 2 Department of Otorhinolaryngology, Clínica Las Condes, Santiago, Chile; 3 Department of Otorhinolaryngology – Head and Neck Surgery, Hospital Clínico Regional Dr. Guillermo Grant Benavente, Concepción, Chile; 4 Department of Basic and Clinical Oncology, Faculty of Medicine, Universidad de Chile, Santiago, Chile

**Keywords:** squamous cell carcinoma of head and neck, quality indicators, health care, patient care team

## Abstract

**Introduction:**

Head and neck cancer are rare and require complex medical and surgical management. Regionalization or centralization of care, defined as the concentration of patients with complex diseases from a specific area in institutions with more experienced and highly functional multidisciplinary teams, may be an alternative to achieve better oncologic outcomes.

**Objective:**

To systematize the current knowledge regarding the centralization of care in head and neck oncology and its consequences in the practice of related surgeries.

**Data Synthesis:**

Currently, there is evidence that this strategy shows better oncologic outcomes in centers with greater volumes, greater adherence to evidence-based clinical guidelines and quality indicators, and a multidisciplinary team in charge of decision-making. The center in Ontario, Canada, is framed as an example of this strategy, achieving improved outcomes while maintaining a high level of quality.

**Conclusion:**

Although more high-quality studies are needed to support this strategy, we believe that the evidence already available is sufficient to consider it a valid option to improve the oncologic outcomes of patients.

## Introduction

Malignant neoplasms represent a tremendous burden on healthcare systems worldwide. According to data published in the fifth edition of the Global Cancer Observatory (GLOBOCAN) for 2020, the incidence of cancer worldwide was estimated to be 19.13 million cases, along with 10.0 million cancer deaths in that year. 1

Head and neck cancers are a heterogeneous group of malignant neoplasms composed of six groups, depending on their origin: endocrine, mucosal, salivary glands, skin (non-melanoma and melanoma), and skull base carcinomas, as well as sarcomas. The most frequent are the mucosal carcinomas derived from the upper aerodigestive tract (UADT), which had an overall incidence of approximately 878 thousand cases in 2020. The second most frequent is thyroid cancer, with an incidence of 586 thousand cases in 2020. 1 Despite the data mentioned above, it must be considered this cancer type is infrequent, taking into account that worldwide, the most frequent one is lung cancer, with an estimated incidence of 2.2 million cases, followed by breast and colorectal cancer, with an incidence of 2.261 million and 1.1 million cases respectively, in 2020. 1

As a result, various public health measures have been adopted to prevent and treat this condition. The objective of this review is to describe one of those strategies, which is the regionalization of patient care.

## Review of Regionalization or Centralization of Health Care

Regionalization or centralization of health care is defined as the act of concentrating cases or patients with diseases in a specific area in institutions with multidisciplinary teams that have greater experience and are highly functional to achieve better results, especially in complex cases that require more resources for treatment. 2 3 About this issue, there are publications that show better overall survival outcomes among patients treated in specialized institutions. 2 4 The following paragraphs review the different key points of regionalization.

### Multidisciplinary Team

The management of patients with head and neck cancer requires consideration of multiple factors, making therapeutic decisions challenging. Among these factors are those related to histology, subsite, staging, age, sex, comorbidities, and psychosocial aspects, all of which must be strictly analyzed before treatment. 5 For this, a multidisciplinary team should be established, composed of a wide range of health professionals so that each case is approached from all angles. This group should include head and neck surgeons, reconstructive surgeons, medical oncologists, radiation oncologists, radiologists, and pathologists, all ideally specialized in the head and neck, in addition to specialists in palliative care, speech therapists, dietitians, physiotherapists, and nurses specialized in head and neck care. Additionally, including computer scientists and biostatisticians would also benefit the team, as they could manage the data from patient care to analyze them and thus improve the decision-making processes, which are fundamental for the performance of clinical audits. For the multidisciplinary team to be efficient and achieve more effective coordination, it should be in tertiary health centers. 6

In this regard, there are publications that show better oncological outcomes when a multidisciplinary team is used in the care of these patients. One of these publications was the study conducted by Friedland et al., who retrospectively analyzed the results of 726 cases of head and neck cancer in the same institution, divided between those who were and weren't managed by a multidisciplinary team. Among their results, it stands out that stage IV patients and those who were managed by a multidisciplinary team had a better overall survival rate at 5 years compared to those who were not managed by a multidisciplinary team, with a hazard ratio (HR) of 0.69, a 95% confidence interval (95%CI) of 0.51 to 0.88 ( *p* = 0.004). Furthermore, greater use of concomitant chemoradiotherapy was observed in the group managed by a multidisciplinary team ( *p* = 0.004). 7 It can be inferred from this study that the improved survival rates in these patients is probably due to better diagnosis, more accurate staging, more efficient therapeutic approach, and better communication between the different specialties. 8

There is no consensus on the exact number of cases to ensure that this team has the necessary experience to deal with this type of rare disease, as this number varies depending on the country involved. For example, in 2004, the United Kingdom's National Health Service recommended a minimum of 100 cases per year per multidisciplinary UADT cancer team, which involves a designated population of over 1 million people per institution. 6

On the other hand, this multidisciplinary team should not only focus on primary treatment but also on subsequent rehabilitation. This rehabilitation team should contain specialized nurses, dietitians, dentists, physiotherapists, speech therapists, occupational therapists, psycho-oncologists and a team of social workers. This is extremely important for achieving complete and adequate management in this population. 6

### Volume-Outcome Association

Some publications show that institutions with a higher volume of patient care have better outcomes. This has been observed especially in the surgical area, specifically in complex surgeries. This was first introduced by Birkmeyer and collaborators in 2002 when they published a study in which they analyzed the associated mortality in six types of cardiovascular and eight types of oncological surgical procedures. With a sample of 2.5 million people, they observed that mortality decreased as the hospital volume increased, which varied widely depending on the type of procedure. 9

This study showed that, for high-risk or complex surgical procedures, treatment in a high-volume hospital would mean a lower probability of postoperative death. However, this depends on the type of intervention the patient undergoes, which implies not only that the “hospital volume” factor influences the best results, but pre-, intra-, and postoperative care also determine the outcome. One of the factors proposed is “surgeon volume”, which is defined as the number of surgeries per year per surgeon, could have a greater incidence in procedures that do not require prolonged postsurgical management. 9 10

Regarding head and neck surgical oncology, studies have shown the association between volume outcomes. In 2014, Eskander et al. published a systematic review and a meta-analysis incorporating a total of 17 studies, which demonstrated that independent to head and neck cancer subsite, volume definition or outcome assessed, high-volume hospitals (HR: 0.886; 95%CI: 0.820–0.956) and high-volume surgeons (HR: 0.767; 95%CI: 0.641–0.919) were associated with longer survival. 11 Subsequently, Eskander et al. evaluated the relationship between the surgical volume of both surgeons and hospitals and overall survival in patients with head and neck cancer, using the Ontario database between 1993 and 2010, with a cohort of 5,720 patients. It was determined that only volume per hospital was associated with that outcome after adjusting the analysis for the variables analyzed (HR: 0.976; 95%CI: 0.955–0.997). An interesting point of this study is that a minimum threshold was estimated to achieve a linear relationship between overall survival and the number of surgeries performed, which was 30 surgeries per year per surgeon, and 75 surgeries per year per institution. 12

### Quality Indicators

To refer to health quality indicators, we use the classic paradigm proposed by Donabedian, 13 which classifies them into structure, process, and outcome indicators. Each type of indicator has a different purpose. For example, to estimate oncological outcomes, such as overall survival or recurrence rate, outcome indicators are the most important, but to assess the quality of health care, the most relevant indicators are process indicators. 14 The indicators related to head and neck cancer care will be discussed below.

First, structural indicators reflect the environment in which healthcare is framed. There are two major factors that determine this type of indicator: personnel and equipment. For instance, the number of health professionals or institutions for a given population, as well as the interdisciplinary network in which they are located. These indicators alone do not guarantee quality and may not even represent good indicators of the care provided. 15

Process indicators are used to evaluate the delivery of health care to the population. They include all the steps in the patients' management trajectory from at the institution or with the professional. 15 For this reason, the best indicators come from evidence or scientific studies in which a given practice results in a favorable outcome (which is known as the *standard of care* ).

### Head and Neck Multidisciplinary Oncology Meeting (Tumor Board)

It is essential that interdisciplinary teams make decisions in complex cases to obtain better results. Therefore, it is necessary to establish a head and neck oncology multidisciplinary team (MDT) that meets on a weekly basis to cover new cases. 6 7 Variations of up to 60% have been documented in both staging and management of these patients in the presence of MDTs, which reflects their importance. 16

Some publications support the hypothesis that MDTs can have an impact on final oncologic outcomes. In a retrospective study by Liu et al., two periods were analyzed in the same center: before the presence of MDT, which included a cohort of 98 patients, and after, which included a cohort of 126 patients, giving a total of 224 participants. The average follow-up was 2.8 years, and most cases were in the advanced stage (68%). It was observed that the specific survival rate (SSR) improved significantly in the after-MDT cohort with 5 years of 75%, compared with 5 years of 52% for the before-MDT cohort. This reflects a significantly lower risk of death, with a hazard ratio (HR) of 0.48. 17 Although the correlation between MDT and better oncologic outcomes is uncertain, we presume it would lead to more accurate diagnosis and staging, which would allow a refinement in the management plan and lead to better results. 18 19 20

### Adherence to clinical practice guidelines

Clinical practice guidelines (CPG) are developed based on evidence using the best information available up to the date of publication. However, their application should be taken with care, considering that the target populations may be different from the studies that present the CPG recommendations. 15 Theoretically, adherence to CPG recommendations would decrease treatment variability and improve evidence-based management. 12

Gourin et al. published a retrospective study, which analyzed the association between adherence to the American National Comprehensive Cancer Network's guidelines, and short- and long-term outcomes in patients diagnosed with laryngeal squamous cell carcinoma (SCC). They demonstrated that high-quality treatment (with greater adherence to clinical guidelines) was associated with a lower probability of weight loss, with an odds ratio (OR) of 0.6 (95%CI: 0.5–0.8), esophageal stricture (OR: 0.5; 95%CI: 0.3–0.8), gastrostomy dependency (OR: 0.5; 95%CI: 0.4–0.7), airway obstruction (OR: 0.7; 95%CI: 0.6–0.9), tracheostomy (OR: 0.5; 95%CI: 0.3–0.7), and pneumonia (OR: 0.7; 95%CI: 0.5–0.9). 21 This is the only publication to date which relates adherence to clinical guidelines with a better clinical outcome.

### Time of initiation of initial and adjuvant treatment

An important issue is the timing of treatment initiation from initial diagnosis. 1 This is due to the rapidly progressive nature of this disease in a complex and functionally vulnerable anatomical area, which directly impacts survival and quality of life. 15 One way to address this situation is to improve referral from primary healthcare and reduce waiting times. For instance, the United Kingdom's National Institute for Health and Care Excellence has been published a CPG for the recognition and referral of suspected cancer, which was updated in 2015, specifying when such patients should be seen by the specialist, which is 14 days from referral from primary care. Despite these well-established waiting times, their true impact is not yet well known. 22

The impact of the waiting time from diagnosis to initiation of treatment has not been properly studied with level I evidence; however, some studies have attempted to define a threshold demonstrating its impact on survival. Murphy et al. published a retrospective study using the National Cancer Database in patients with head and neck SCC who received treatment with curative intent. They analyzed 51,655 patients and determined that a treatment initiation time of 61 to 90 days independently increased the risk of mortality (HR: 1.13) when compared with less than 30 days. They also determined threshold ranges, between 46 to 52 days and between 62 and 67 days, with a more consistent increase in the risk of death beyond 60 days. 23

Other relevant times are the time between surgery and the start of adjuvant radiotherapy when indicated, and between surgery and completion of adjuvant therapy when necessary, the latter of which is also known as package time. 24 Despite some heterogeneity in the definition, it is generally accepted that the time between surgery and adjuvant therapy should be limited to less than or equal to 6 weeks, which has shown to have an impact on reducing the risk of death in patients treated surgically for head and neck SCC. 24 On the other hand, the package time is not well established and has a variable definition ranging from 77 to 100 or more days. 25 Although its impact is not well established, observational studies have shown a lower survival in patients with a package time of more than 12 weeks. 25

### Surgical oncology and quality criteria

Regarding surgical quality criteria, it is important to note that there are currently no quality criteria adopted by any national quality institution to evaluate this area. However, attempts have been made to determine the indicators that would have a concrete impact on oncologic outcomes.

Cramer et al. published in 2017 a study where they analyzed the impact on oncologic outcomes of five quality indicators in patients with head and neck SCC who underwent surgical treatment, specifically on overall survival, using the NCDB database between 2004 and 2014. These five indicators were: negative surgical oncologic margins; neck dissection with a number greater than or equal to 18 lymph nodes; access to adjuvant radiotherapy, if indicated; access to adjuvant chemoradiotherapy, if indicated; and initiation of adjuvant therapy within a 6-week period following surgery. Management was considered high-quality if it met more than 75% of the indicators. A total of 76,853 patients were included in this study, and compliance with these factors on an individual basis significantly reduced the risk of death. It was also determined that academic and/or high-volume centers, defined as centers with > 160 surgeries in a 10-year period, had better oncologic results than nonacademic and low-volume centers. When analyzing those with high-quality management, the adjusted risk of death was reduced by 19%, being more likely to receive high-quality management in higher-volume centers. 20

However, it is interesting to note that this type of metric is easily measurable and can be evaluated at a national or regional level to be considered a quality criterion in oncologic surgery. On the other hand, there have been attempts to determine quality indicators in the case of reconstruction with free microvascular flaps in head and neck surgery.

Eskander et al. 26 published a retrospective study in 2018 that analyzed 515 patients who underwent head and neck reconstruction using free flaps at Ohio State University to determine predictors for prolonged hospital admission, readmission within 30 days, and readmission to the operating room within 30 days. The predictors found were tumor location in the oral cavity and pharynx, blood transfusion, diabetes mellitus, or any type of complication (both medical and surgical) for prolonged hospital admission. Furthermore, absence of preoperative evaluation, presence of any type of postoperative complication for hospital readmission, and advanced age were predictors for readmission to the operating room. Thus, efforts should be made to find early predictors of complications and the preoperative clinical evaluation should be a quality indicator, due to its impact on early readmission.

The last group is the outcome indicators, which refer to the evaluation of the final result of health care. For this reason, historically, the most important indicator in oncology has been overall survival. At this point, it is important to point out that, despite improvements on technology, especially regarding treatment with radio or chemoradiotherapy, the focus was not only on improving survival as such, but also on reducing both their acute and late toxicity. 15

Therefore, another important topic regarding outcome indicators is the evaluation of adverse events to oncologic treatments in head and neck. This is of great importance when considering that related treatments correspond to the 14th most common cause of disease burden worldwide. 27 In the particular case of head and neck cancer, these adverse events, related both to surgical treatment and to chemo or radiotherapy, are well known and should be systematically reported and recorded in order to determine deficits and possible strategies to improve the results of a particular department. Among the adverse effects are those derived from ablative surgery, such as functional difficulties, scarring, cosmetic and communication alterations, as well as others derived from radio or chemotherapy such as fibrosis, osteoradionecrosis, or xerostomia. 15

The concept that encompasses the evaluation of quality of life and functional aspects, as well as mental health and the management of complications, are the “survivorship” outpatient clinics, where patients are periodically evaluated in parallel to oncologic controls to evaluate and manage the aspects described above.

### Compliance to Quality Indicators

The above-mentioned indicators have a concrete oncological impact on the management and outcomes of patients with head and neck cancer. This has been demonstrated in other surgical areas, where the adoption of quality indicators in different centers improves surgical outcomes, reducing the number of complications and also reducing the costs associated with health care. 28 Therefore, in 2007, the American Head and Neck Society (AHNS) approved the first group of quality indicators for oral cancer and, in 2009, for laryngeal cancer. Both sets of indicators are very similar and include aspects such as documentation of pathological anatomy, diagnostic stage, tobacco cessation counseling, interdisciplinary evaluation by surgical oncology, medical oncology, and radiation oncology, and standardized follow-up for early detection of recurrence, second primaries, complications, and hypothyroidism in the case of radiotherapy. 29

In head and neck oncology, the RARECAREnet project working group have elaborated, through a panel of multidisciplinary experts in the management of this type of cancer, 11 quality indicators that address both diagnosis and treatment care for patients with head and neck cancer, with a focus on surgery and radiotherapy, not addressing chemotherapy. The same working group conducted an observational study incorporating the cancer registries of four European countries (Italy, Holland, Slovenia, and Scotland) to evaluate their degree of compliance for each quality indicator, specifically SCC of the larynx, oral cavity, oropharynx, and hypopharynx. Regarding the oncological management it showed that adherence to CPG was high in patients with localized disease with a range of 72 to 79%. However, it was low in advanced disease with a range of 19 to 44. Regarding time to adjuvant radiotherapy, most patients started adjuvant radiotherapy 8 weeks after surgery, ranging from 52% to 79%. 18

Currently, more studies are needed to determine whether greater compliance with these quality indicators could improve oncological outcomes in these patients. However, the previously mentioned study shows a room for improvement in this issue.

### Benefits in Resource Management

Health care systems are currently seeking strategies to increase the value of the services provided and the arguments explained above favor the adoption of public policies that lead to the allocation of greater efforts to centralize the treatment of these more complex conditions. Such policies have been implemented in several countries, including the United Kingdom, Netherlands, Canada, and Australia, which have made efforts to consolidate the care of complex cases in high-volume institutions in order to improve quality and reduce associated costs. 30 31 32 However, there are no economic studies, nor are there any with a high level of evidence to support this assertion.

### The Ontario Experience

The province of Ontario, Canada, is one of the most important examples of the regionalization of cancer treatment. Since 1998, a surgical oncology program has been in place under the Cancer Care Ontario (CCO), aiming to address the high variations in diagnostic and surgical procedures performed on cancer patients in Ontario in the face of the limited evidence available in the early 1990s. 2

This program led to the formation of surgical oncology departments in regional cancer centers, to promote referrals to these centers. Additionally, this program has promoted strategies to improve surgical care, developing evidence-based CPG and establishing standards for surgical procedures especially for those centers far from the large cities of the province. Among the strategies adopted, the creation of “Centers of Expertise” for highly complex surgeries stands out, in order to ensure a better outcome for patients 2 . In addition to the strategies adopted to reduce surgical service delivery time. A waiting time strategy (WTS) for oncologic diseases was created and led by the CCO. This established the maximum waiting times for certain groups of diseases, defining different objectives according to priority levels for different diseases. 2

Specifically, in patients with head and neck cancer, most ablative surgeries are performed in experienced centers, accounting for most patients with oral cavity (90%), laryngeal/hypopharyngeal (98%), and salivary gland cancers (57%). 33 Regarding their oncologic outcomes, there was a clear improvement in the institution's volume after controlling for clustering and patient/treatment covariates, with a 2.4% decrease in the HR of death for every 25 additional cases. 10 These results support the management strategies adopted by the province.

## Conclusion

The centralization of health care in complex diseases, especially in oncology, has been shown to improve outcomes. This strategy could be associated with greater experience and coordination between oncologic and allied health teams. Moreover, it is associated with the advantages of high-volume institutions, which are more likely to have a better-trained multidisciplinary team and better structural elements to resolve the inherent complexities of these scenarios. These better outcomes lead to longer survival and potentially better quality of life for patients.

While it is true that this may lead to a decrease in the volume and experience of lower-volume hospitals, in rare diseases such as head and neck cancers, it is imperative to aim for the best quality of treatment and possible outcomes. Although more high-quality studies are needed to support this strategy, we believe that the evidence already available is sufficient to consider it a valid option to improve the oncologic outcomes of patients.

## References

[1] Sung H Ferlay J Siegel R L Global Cancer Statistics 2020: GLOBOCAN Estimates of Incidence and Mortality Worldwide for 36 Cancers in 185 Countries CA Cancer J Clin 2021 71 03 209 249 https://pubmed.ncbi.nlm.nih.gov/33538338/cited 2023Dec27 [Internet] 33538338 10.3322/caac.21660

[2] Sullivan T Irish JBuilding the Ontario Surgical Oncology Program.https://doi.org/101177/0840470417729171[Internet]. 2017 Dec 12 [cited 2023 Dec 27];31(1):22–5. Available from:https://journals.sagepub.com/doi/10.1177/0840470417729171 10.1177/0840470417729171 29231070

[3] Disparities in care point to need for complex cancer surgical centres – Canadian Partnership Against Cancer [Internet]. [cited 2023 Dec 27]. Available from:https://www.partnershipagainstcancer.ca/news-events/news/article/disparities-in-care-point-to-need-for-complex-cancer-surgical-centres/

[4] Hollenbeck B K Miller D C Wei J T Montie J E Regionalization of care: centralizing complex surgical procedures Nat Clin Pract Urol 2005 2 10 461 https://pubmed.ncbi.nlm.nih.gov/16474601/cited 2023Dec27 [Internet] 16474601 10.1038/ncpuro0322

[5] RARECAREnet Working Group Orlandi E Alfieri S Simon C Trama A Licitra L Treatment challenges in and outside a network setting: Head and neck cancers Eur J Surg Oncol 2019 45 01 40 45 https://pubmed.ncbi.nlm.nih.gov/29478741/cited 2023 Dec 27 [Internet] 29478741 10.1016/j.ejso.2018.02.007

[6] James N Hartley A Improving outcomes in head and neck cancer Clin Oncol (R Coll Radiol) 2003 15 05 264 265 https://pubmed.ncbi.nlm.nih.gov/12924457/cited 2023 Dec 27 [Internet] 12924457 10.1016/s0936-6555(03)00167-5

[7] Friedland P L Bozic B Dewar J Kuan R Meyer C Phillips M Impact of multidisciplinary team management in head and neck cancer patients Br J Cancer 2011 104 08 1246 1248 https://pubmed.ncbi.nlm.nih.gov/21448166/cited 2023 Dec 27 [Internet] 21448166 10.1038/bjc.2011.92 PMC3078600

[8] Tsai W C Kung P T Wang S T Huang K H Liu S A Beneficial impact of multidisciplinary team management on the survival in different stages of oral cavity cancer patients: results of a nationwide cohort study in Taiwan Oral Oncol 2015 51 02 105 111 https://pubmed.ncbi.nlm.nih.gov/25484134/cited 2023 Dec 27 [Internet] 25484134 10.1016/j.oraloncology.2014.11.006

[9] Birkmeyer J D Siewers A E Finlayson E VA Hospital volume and surgical mortality in the United States N Engl J Med 2002 346 15 1128 1137 https://pubmed.ncbi.nlm.nih.gov/11948273/cited 2023 Dec 27 [Internet] 11948273 10.1056/NEJMsa012337

[10] Eskander A Irish J Groome P A Volume-outcome relationships for head and neck cancer surgery in a universal health care system Laryngoscope 2014 124 09 2081 2088 https://pubmed.ncbi.nlm.nih.gov/24706437/cited 2023 Dec 27 [Internet] 24706437 10.1002/lary.24704

[11] Eskander A Merdad M Irish J C Volume-outcome associations in head and neck cancer treatment: a systematic review and meta-analysis Head Neck 2014 36 12 1820 1834 https://pubmed.ncbi.nlm.nih.gov/24123512/cited 2023 Dec 27 [Internet] 24123512 10.1002/hed.23498

[12] Eskander A Goldstein D P Irish J C Health Services Research and Regionalization of Care-From Policy to Practice: the Ontario Experience in Head and Neck Cancer Curr Oncol Rep 2016 18 03 19 https://pubmed.ncbi.nlm.nih.gov/26869188/cited 2023 Dec 27 [Internet] 26869188 10.1007/s11912-016-0500-6

[13] Donahedian. La definición de calidad y enfoques para su evaluación. Vol 1. Exploraciones en Evaluación y Monitoreo de la Calidad. Health Administration Press: 2017;1(ISBN: 9780914904489.):1988

[14] Chen A Y Quality initiatives in head and neck cancer Curr Oncol Rep 2010 12 02 109 114 https://pubmed.ncbi.nlm.nih.gov/20425595/cited 2023 Dec 27 [Internet] 20425595 10.1007/s11912-010-0083-6

[15] Takes R P Halmos G B Ridge J A Value and Quality of Care in Head and Neck Oncology Curr Oncol Rep 2020 22 09 92. Available from: /pmc/articles/PMC7351804/32651680 10.1007/s11912-020-00952-5 PMC7351804

[16] Bergamini C Locati L Bossi P Does a multidisciplinary team approach in a tertiary referral centre impact on the initial management of head and neck cancer? Oral Oncol 2016 54 54 57 https://pubmed.ncbi.nlm.nih.gov/26774920/cited 2023 Dec 27 [Internet] 26774920 10.1016/j.oraloncology.2016.01.001

[17] Liu J C Kaplon A Blackman E Miyamoto C Savior D Ragin C The impact of the multidisciplinary tumor board on head and neck cancer outcomes Laryngoscope 2020 130 04 946 950. Available from: /pmc/articles/PMC7868105/31095740 10.1002/lary.28066 PMC7868105

[18] Trama A Botta L Foschi R Quality of Care Indicators for Head and Neck Cancers: The Experience of the European Project RARECAREnet Front Oncol 2019 9 837. Available from: /pmc/articles/PMC6722861/31555591 10.3389/fonc.2019.00837 PMC6722861

[19] van Overveld L FJ Braspenning J CC Hermens R PMG Quality indicators of integrated care for patients with head and neck cancer Clin Otolaryngol 2017 42 02 322 329 https://pubmed.ncbi.nlm.nih.gov/27537106/cited 2023 Dec 27 [Internet] 27537106 10.1111/coa.12724

[20] Cramer J D Speedy S E Ferris R L Rademaker A W Patel U A Samant S National evaluation of multidisciplinary quality metrics for head and neck cancer Cancer 2017 123 22 4372 4381 https://pubmed.ncbi.nlm.nih.gov/28727137/cited 2023 Dec 27 [Internet] 28727137 10.1002/cncr.30902

[21] Gourin C G Starmer H M Herbert R J Quality of care and short- and long-term outcomes of laryngeal cancer care in the elderly Laryngoscope 2015 125 10 2323 2329 https://pubmed.ncbi.nlm.nih.gov/26010671/cited 2023 Dec 27 [Internet] 26010671 10.1002/lary.25378

[22] Langton S Siau D Bankhead C Two-week rule in head and neck cancer 2000-14: a systematic review Br J Oral Maxillofac Surg 2016 54 02 120 131 https://pubmed.ncbi.nlm.nih.gov/26795572/cited 2023 Dec 27 [Internet] 26795572 10.1016/j.bjoms.2015.09.041

[23] Murphy C T Galloway T J Handorf E A Survival Impact of Increasing Time to Treatment Initiation for Patients With Head and Neck Cancer in the United States J Clin Oncol 2016 34 02 169 178. Available from: /pmc/articles/PMC4858932/26628469 10.1200/JCO.2015.61.5906 PMC4858932

[24] Graboyes E M Kompelli A R Neskey D M Association of Treatment Delays With Survival for Patients With Head and Neck Cancer: A Systematic Review JAMA Otolaryngol Head Neck Surg 2019 145 02 166 177 https://pubmed.ncbi.nlm.nih.gov/30383146/cited 2023 Dec 27 [Internet] 30383146 10.1001/jamaoto.2018.2716 PMC6494704

[25] Goel A N Frangos M I Raghavan G The impact of treatment package time on survival in surgically managed head and neck cancer in the United States Oral Oncol 2019 88 39 48 https://pubmed.ncbi.nlm.nih.gov/30616795/cited 2023 Dec 27 [Internet] 30616795 10.1016/j.oraloncology.2018.11.021

[26] Eskander A Kang S Y Tweel B Quality Indicators: Measurement and Predictors in Head and Neck Cancer Free Flap Patients Otolaryngol Head Neck Surg 2018 158 02 265 272 https://pubmed.ncbi.nlm.nih.gov/29293404/cited 2023 Dec 27 [Internet] 29293404 10.1177/0194599817742373

[27] Jha A K Larizgoitia I Audera-Lopez C Prasopa-Plaizier N Waters H Bates D W The global burden of unsafe medical care: analytic modelling of observational studies BMJ Qual Saf 2013 22 10 809 815 https://pubmed.ncbi.nlm.nih.gov/24048616/cited 2023 Dec 27 [Internet] 10.1136/bmjqs-2012-001748 24048616

[28] Hall B L Hamilton B H Richards K Bilimoria K Y Cohen M E Ko C Y Does surgical quality improve in the American College of Surgeons National Surgical Quality Improvement Program: an evaluation of all participating hospitals Ann Surg 2009 250 03 363 376 https://pubmed.ncbi.nlm.nih.gov/19644350/cited 2023 Dec 27 [Internet] 19644350 10.1097/SLA.0b013e3181b4148f

[29] The development of quality of care measures for oral cavity cancer Arch Otolaryngol Head Neck Surg 2008 134 06 672 https://pubmed.ncbi.nlm.nih.gov/18559740/cited 2023 Dec 27 [Internet] 18559740 10.1001/archotol.134.6.672

[30] Chen M M Megwalu U C Liew J Sirjani D Rosenthal E L Divi V Regionalization of head and neck cancer surgery may fragment care and impact overall survival Laryngoscope 2019 129 06 1413 1419 https://pubmed.ncbi.nlm.nih.gov/30152007/cited 2023 Dec 27 [Internet] 30152007 10.1002/lary.27440

[31] Roman B R Awad M I Patel S G Defining value-driven care in head and neck oncology Curr Oncol Rep 2015 17 01 424 https://pubmed.ncbi.nlm.nih.gov/25416318/cited 2023 Dec 27 [Internet] 25416318 10.1007/s11912-014-0424-y

[32] EUROCARE Working Group Gatta G Botta L Sánchez M J Anderson L A Pierannunzio D Licitra L Prognoses and improvement for head and neck cancers diagnosed in Europe in early 2000s: The EUROCARE-5 population-based study Eur J Cancer 2015 51 15 2130 2143 https://pubmed.ncbi.nlm.nih.gov/26421817/cited 2023 Dec 28 [Internet] 26421817 10.1016/j.ejca.2015.07.043

[33] ICES | Head and Neck Cancer Surgery in Ontario, 2003-2010: An ICES Atlas [Internet]. [cited 2023 Dec 27]. Available from:https://www.ices.on.ca/publications/atlases/head-and-neck-cancer-surgery-in-ontario-2003%E2%80%922010-an-ices-atlas/

